# P-1904. Training the Next Generation of Tropical Medicine Specialists: A 12-Year Analysis of One Center’s Diploma of Tropical Medicine (DTM) Trainee Cohorts

**DOI:** 10.1093/ofid/ofaf695.2073

**Published:** 2026-01-11

**Authors:** Livia Frost, Austin Huang, Eva Clark, Megan M Duffey, Jill E Weatherhead

**Affiliations:** Baylor College Of Medicine, Houston, TX; Baylor College of Medicine, Houston, Texas; Baylor College of Medicine, Houston, Texas; Baylor College of Medicine, Houston, Texas; Baylor College of Medicine, Houston, Texas

## Abstract

**Background:**

U.S. Infectious Diseases (ID) fellowship programs are facing declining applicants; 2025 NRMP Specialties Matching Service data showed nearly half of ID programs had unfilled positions. This shortage is particularly concerning given ID specialists’ importance in tropical and vector-borne disease hotspots, areas expanding due to climate change, urbanization, travel, and undervaccination. Baylor College of Medicine (BCM)’s Diploma of Tropical Medicine (DTM) provides training in tropical bacteriology, virology, parasitology, and public health. This study’s objective was to describe the BCM DTM trainee cohorts since the course was founded in 2011.

**Figure:**
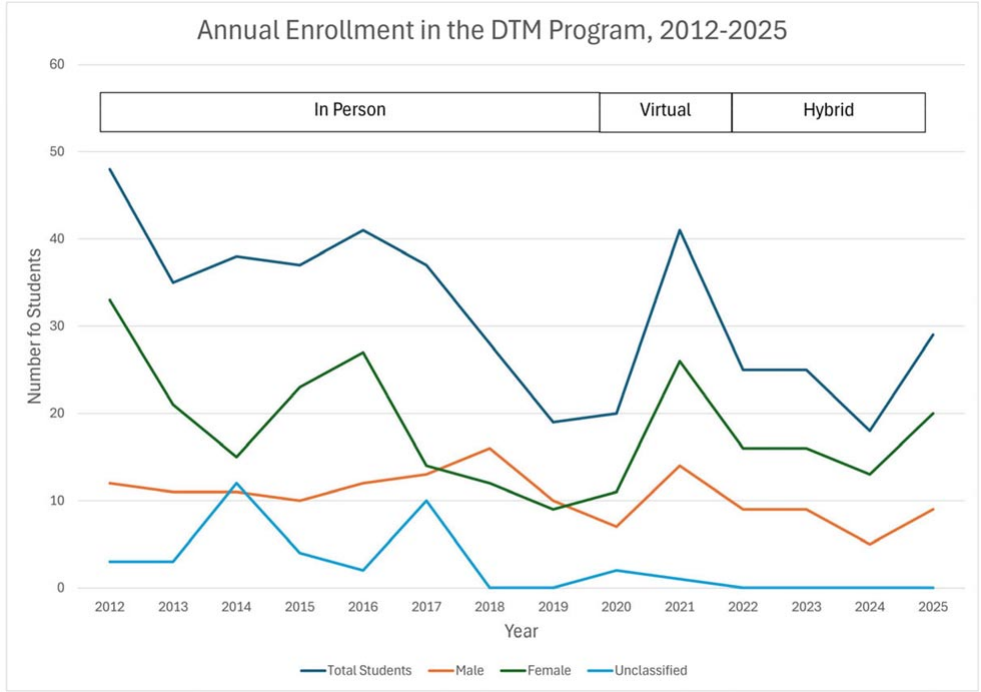

**Figure:**
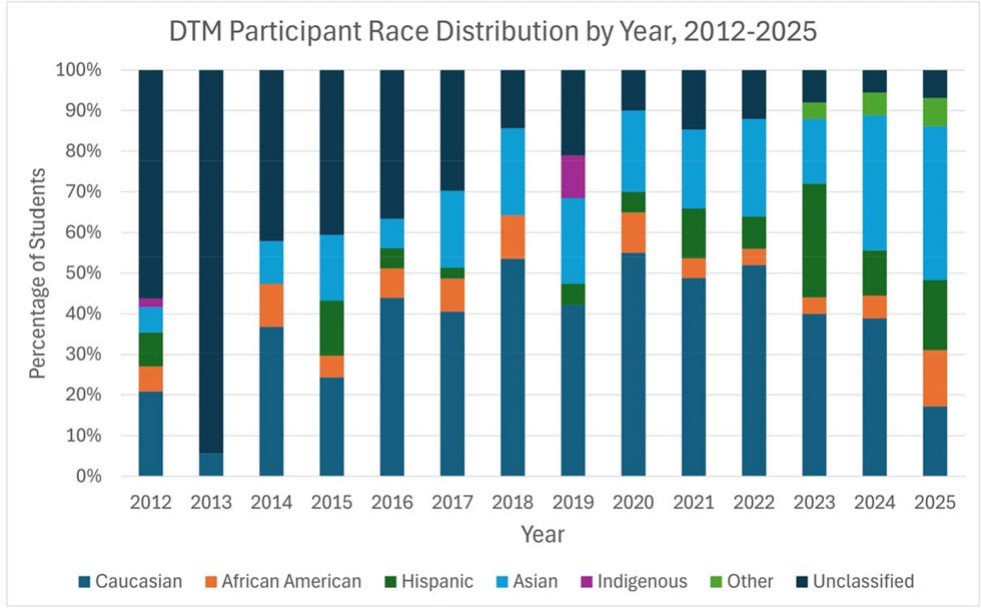

**Methods:**

We utilized descriptive statistics to analyze de-identified trainee data from BCM’s DTM program between 2012–2025, including demographics, course completion, and Certificate of Knowledge in Clinical Tropical Medicine and Travelers’ Health (CTropMedÒ) exam outcomes.

**Figure:**
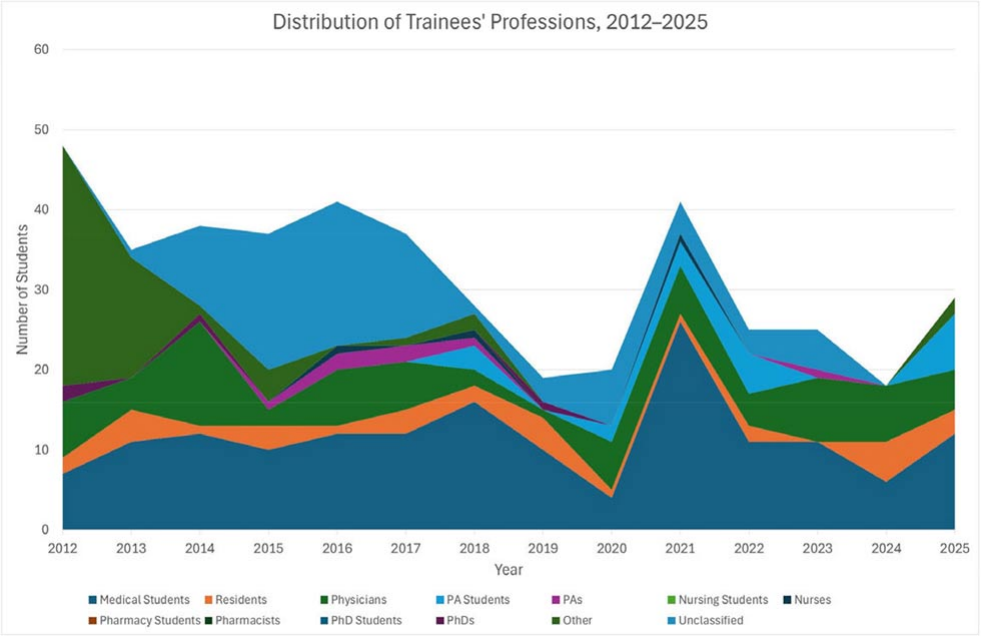

**Figure:**
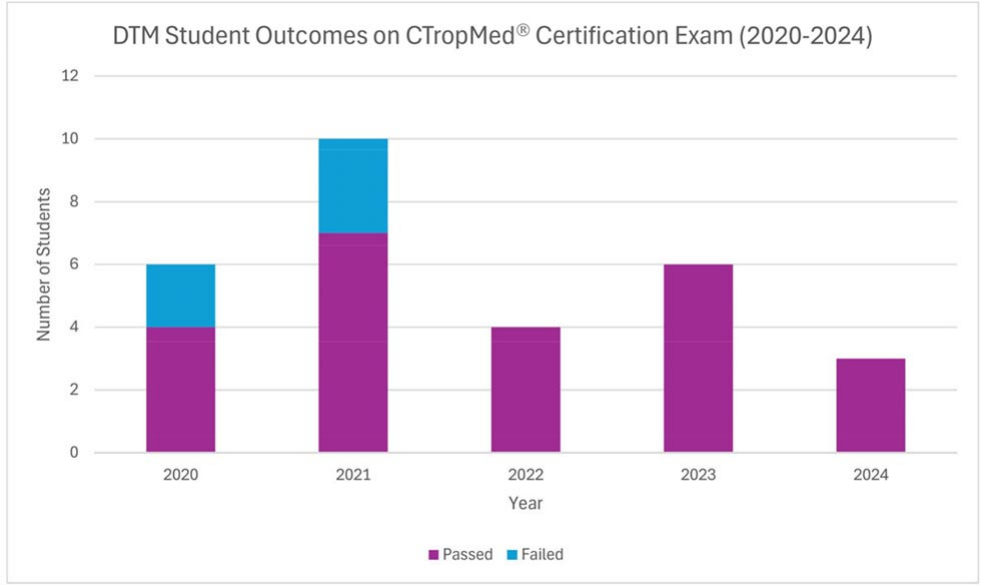

**Results:**

We evaluated 441 DTM trainees (Figure 1). Enrollment averaged 39 trainees/year between 2012-2017, then declined to 22/year between 2018-2020, and rebounded to 29 trainees in 2025. Most trainees (58%/year) were women. 2025 marked the first year that non-White groups (Asian 38%, Hispanic 17%, Black 14%) comprised the majority (69%) of trainees (Figure 2). Most trainees were medical students (36%) or physicians (32%), while physician assistant (PA) students emerged as a growing cohort (24% of the 2025 class, from 0% in 2011-2017) (Figure 3). DTM completion rates increased from a pre-2021 average of 44%/year to 76-80% in 2022-2024. Between 2020–2024, CTropMedÒ exam participation among DTM students remained stable (3–10 annually), with pass rates improving from 70% in 2020–2021 to 100% in 2022–2024 (Figure 4).

**Conclusion:**

These trends demonstrate BCM DTM's success in attracting diverse trainees and training them in tropical medicine. Through its hybrid model integrating didactic training, hands-on labs, and mentorship, the BCM DTM can facilitate early interest in ID and equips clinicians for emerging tropical disease challenges.

**Disclosures:**

Eva Clark, MD, PhD, American Academy of HIV Medicine: Honoraria|McGraw Hill: Royalties|Novartis: Grant/Research Support|Wolters Kluwer: Royalties

